# Unilateral Huge Hydronephrosis Necessitating Fetal Interventions

**Published:** 2013-04-01

**Authors:** Ayşenur Cerrah Celayir, Zeki Şahinoğlu, Selçuk Selçuk, Serdar Moralıoğlu, Oktav Bosnalı

**Affiliations:** Department of Pediatric Surgery, Zeynep Kamil Maternity and Children’s Training and Research Hospital İstanbul, Türkiye; 1 Department of Obstetrics and Gynecology, Zeynep Kamil Maternity and Children’s Training and Research Hospital İstanbul, Türkiye

**Dear Sir**

Fetal intervention for obstructive uropathy was first performed at the University of California, San Francisco in 1981 [1]. Since then diagnostic criteria for fetal intervention have been laid down to assist in proper patient selection [1-3]. Unilateral fetal hydronephrosis doesn’t require prenatal intervention; but prenatal intervention might be required in selected cases, especially when hydronephrosis compresses adjacent organs [4].

A 26-year-old pregnant woman was referred to our prenatal center for a fetal abdominal cystic mass with severe oligohydramnios detected at 27th week of gestation. Prenatal scan showed a single male fetus with a cystic avascular mass (74×84 mm) located in the left hemi abdomen pushing left hemi diaphragm (Fig. 1). 

**Figure:**
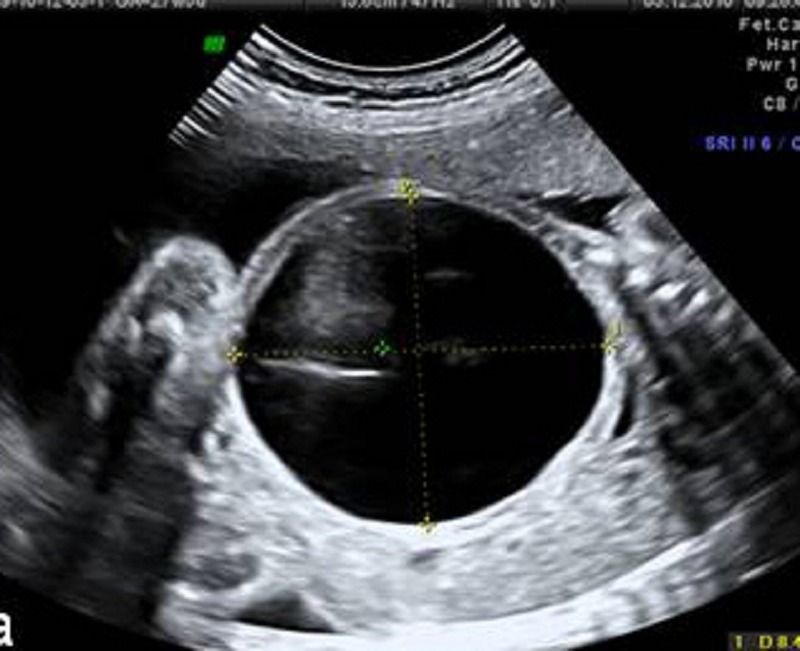
Figure 1: A huge cyst in fetus compressing surrounding structures

Umbilical artery Doppler velocity indices were normal. There was no sign of fetal hydrops. The left kidney could not be visualized; however the right kidney and bladder were normal. Ultrasound guided cyst aspiration was performed which revealed left hydronephrotic kidney. Our prenatal diagnosis was a left huge hydronephrosis or an urinoma secondary to pelvi-ureteric junction (PUJ) obstruction. The urinary electrolytes were in normal ranges (sodium concentration &100 mEq/L, chloride concentration &90 mEq/L, and osmolarity level &210 mOsm). After prenatal counseling of parents by a multidisciplinary team, insertion of a percutaneous catheter into the left renal pelvis was decided to avoid excessive pressure on the left pulmonary parenchyma via elevated left diaphragm. A double pigtail catheter (Cook Medical, Harrison Bladder Catheter) was successfully inserted under ultrasound guidance. Patient was discharged after 24 hours uneventful observation. Antenatal follow-up was uneventful during the rest of the pregnancy. At term, a baby with a catheter tip located on the left upper abdomen was delivered (Fig. 2). 

**Figure:**
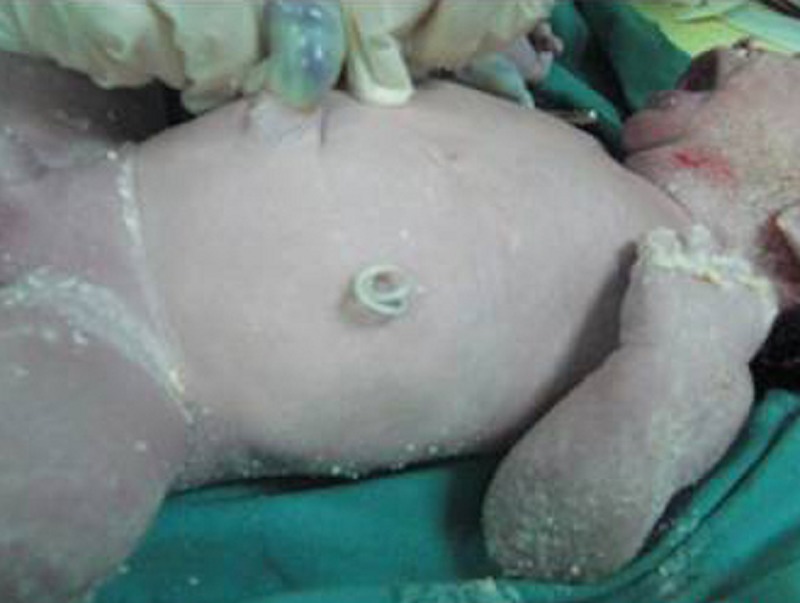
Figure 2: Newborn with catheter tip

APGAR scores were 8 and 10 at the first and fifth minutes. Postnatal ultrasound showed right kidney 51 mm in size with 11 mm parenchyma; and left kidney was 41 mm with parenchyma measuring 9.6 mm, and pelvic AP diameter was 9 mm. Voiding cystourethrogram revealed normal bladder functions (filling and emptying); vesicoureteral reflux and postvoid residue were absent. The kidney catheter was removed on third days of life. The differential function of the left kidney was less than 20% of total renal function by Tc-MAG3 performed one month later (Fig. 3).

**Figure:**
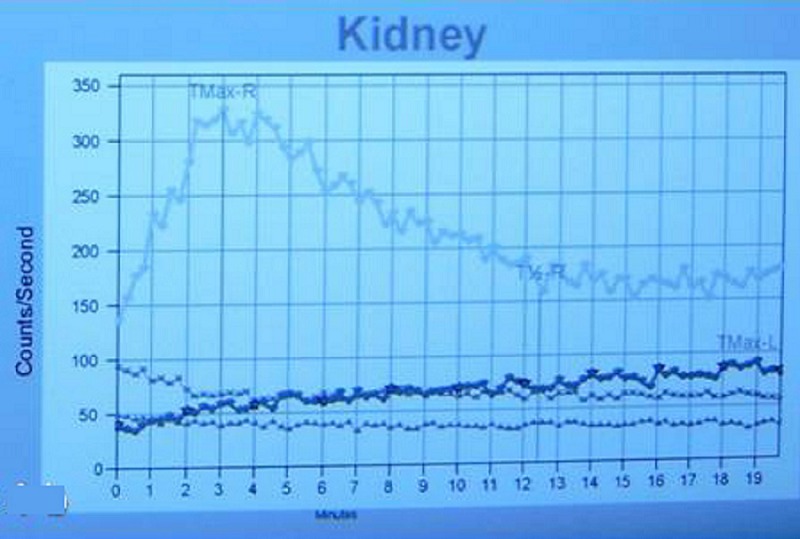
Figure 3: Tc-MAG3 showing left kidney function less than 20% of total renal function

At six months, the differential function of the left kidney was reduced to less than 10%. Bladder compliance (volume/pressure) and total bladder capacity were found normal by Urodynamics. One year later, a progressive decrease of the left kidney functions was demonstrated by scintigraphy. Left nephrectomy is planned after discussion with parents.

Early detection of obstructive uropathy and fetal intervention may avoid renal dysplasia and pulmonary hypoplasia secondary to oligohydramnios. Fetal intervention has limited scope in late presenting cases where renal dysplasia have already occurred [1-3]. Fetal interventions are not recommended in case of unilateral hydronephrosis in presence of normal contralateral kidney. Moreover, fetal shunting procedures do not improve renal outcome [5] especially in late presenting cases like the index case, therefore antenatal intervention has been reserved only in those cases of large urinoma/hydronephrosis that seem to interfere with the functions of other organ systems like pulmonary hypoplasia secondary to diaphragmatic elevation [4]. In our case, insertion of fetal catheter into unilateral huge hydronephrosis did not improve renal function; however respiratory function presumably improved.

## Footnotes

**Source of Support:** Nil

**Conflict of Interest:** None
